# Stable association of RNAi machinery is conserved between the cytoplasm and nucleus of human cells

**DOI:** 10.1261/rna.056499.116

**Published:** 2016-07

**Authors:** Roya Kalantari, Jessica A. Hicks, Liande Li, Keith T. Gagnon, Viswanadham Sridhara, Andrew Lemoff, Hamid Mirzaei, David R. Corey

**Affiliations:** 1 Department of Pharmacology, Department of Biochemistry, University of Texas Southwestern Medical Center, Dallas, Texas 75390, USA; 2 Department of Biochemistry and Molecular Biology, Southern Illinois University, Carbondale, Illinois 62901, USA; 3 Department of Biochemistry, University of Texas Southwestern Medical Center, Dallas, Texas 75390, USA

**Keywords:** Argonaute2, RNAi, proteomics, mass spectrometry, nucleus

## Abstract

Argonaute 2 (AGO2), the catalytic engine of RNAi, is typically associated with inhibition of translation in the cytoplasm. AGO2 has also been implicated in nuclear processes including transcription and splicing. There has been little insight into AGO2's nuclear interactions or how they might differ relative to cytoplasm. Here we investigate the interactions of cytoplasmic and nuclear AGO2 using semi-quantitative mass spectrometry. Mass spectrometry often reveals long lists of candidate proteins, complicating efforts to rigorously discriminate true interacting partners from artifacts. We prioritized candidates using orthogonal analytical strategies that compare replicate mass spectra of proteins associated with Flag-tagged and endogenous AGO2. Interactions with TRNC6A, TRNC6B, TNRC6C, and AGO3 are conserved between nuclei and cytoplasm. TAR binding protein interacted stably with cytoplasmic AGO2 but not nuclear AGO2, consistent with strand loading in the cytoplasm. Our data suggest that interactions between functionally important components of RNAi machinery are conserved between the nucleus and cytoplasm but that accessory proteins differ. Orthogonal analysis of mass spectra is a powerful approach to streamlining identification of protein partners.

## INTRODUCTION

Argonaute (AGO) proteins are key components of RNA interference (RNAi) (Meister 2013) and bind directly to miRNAs and siRNAs to form complexes that regulate gene expression. AGO proteins are conserved and are expressed ubiquitously in animals, plants, and yeast. There are four AGO variants in human cells (AGO1–4) (Schürmann et al. 2013). AGO2 is the only AGO protein to retain catalytic activity, and when combined with an siRNA, can reconstitute RNAi in defined cell-free systems (Liu et al. 2004).

AGO2 is essential for post-translational silencing of mRNA in the cytoplasm of mammalian cells and many studies have focused on this cytoplasmic role. Additional factors known to be involved in cytoplasmic RNAi include TNRC6A (GW182) (Jakymiw et al. 2005; Eulalio et al. 2008; Nishi et al. 2013; Pfaff and Meister 2013), Dicer (Bernstein et al. 2001; Ha and Kim 2014), and TAR RNA binding protein (TRBP) (Daniels and Gatignol 2012; Wilson and Doudna 2013; Takahashi et al. 2014). TNRC6A influences the subcellular localization of AGO2 and is critical for miRNA-guided silencing. Dicer is an endoribonuclease that is responsible for processing pre-miRNAs to form mature, double-stranded miRNAs. Dicer and TRBP bind double-stranded miRNAs and assist loading of the guide strand into AGO.

While most attention has focused on RNAi in the cytoplasm, AGO proteins are also active in the nucleus (Gagnon and Corey 2012; Gagnon et al. 2014a; Schraivogel and Meister 2014; Schraivogel et al. 2015). AGO2 can act in conjunction with guide RNAs to regulate transcription (Chu et al. 2010; Matsui et al. 2013) and splicing (Ameyar-Zazoua et al. 2012; Liu et al. 2015) in human cells. While RNAi can be active in cell nuclei, there are also significant differences. Trax, Translin, and Hsp90, factors that assist guide strand loading onto AGO2, are absent in cell nuclei and loading does not occur in nuclear extracts (Gagnon et al. 2014a).

Understanding the role of RNAi in cell nuclei will require information about nuclear protein–protein interactions and how they compare with those occurring during cytoplasmic RNAi. While no study has specifically examined global nuclear interactions, several proteomic studies have investigated potential interactions of AGO2 in whole cell, cytoplasm, and chromatin (Meister et al. 2005; Höck et al. 2007; Robb and Rana 2007; Landthaler et al. 2008; Weinmann et al. 2009; Ameyar-Zazoua et al. 2012; Frohn et al. 2012; Carissimi et al. 2015).

Tuschl and coworkers examined interactions of Flag-tagged AGO1 and AGO2 (Meister et al. 2005). They used gel electrophoresis to purify specific bands and observed interactions with TNRC6B and MOV10. In a subsequent study, Meister used cells expressing Flag-AGO1 or Flag-AGO2 and immunoprecipitated tagged AGO1 and AGO2 from whole cell extracts (Höck et al. 2007). This study identified a large number of candidate interacting proteins including several involved in RNA metabolism. Robb and Rana (2007) took an alternative approach using a biotinylated siRNA to isolate Ago complexes prior to mass spectrometry. Two purified bands were analyzed and the proteins RHA and HSP 90b were identified. Tuschl subsequently examined interactions of Flag-tagged Ago1-4 in whole cell extract (Landthaler et al. 2008). This study found a wide range of interacting proteins including Dicer, YBX1, RPS6, and RPL7. Meister looked for common interacting factors of all four Flag-tagged AGO proteins and identified Importin 8 (Weinmann et al. 2009). Finally, Meister's group used whole cell extract from mouse embryonic fibroblast cells and found that AGO2 can interact with RNA in the absence of miRNA (Frohn et al. 2012).

One study has focused on examining proteins within chromatin. Harel-Bellan and colleagues examined interactions between proteins with Flag-AGO1 and Flag-AGO2 and identified SRSF splicing factors as candidate interacting partners (Ameyar-Zazoua et al. 2012). Another recent study by Carissimi et al. (2015) in whole cell extracts reported interactions between endogenous AGO2 and SWI/SNF proteins normally found in the nucleus.

These prior studies have provided important information about candidate interacting proteins, but gaps remain in our understanding of factors that interact with AGO2 to facilitate function in both cytoplasm and nuclei. Modern mass spectrometry is sensitive and detects potential interactions with many proteins. Discriminating between real and artifactual interactions is a major challenge to accurate analysis.

One limitation of prior work is that most studies relied on overexpressed Flag-tagged AGO2. The use of a tagged protein increases efficiency of immunoprecipitations, but using a tag affects interactions with other proteins, and overexpression can cause nonphysiologic interactions and increase identification of false positives (Oeffinger 2012). None of these previous studies compared interactions of Flag-tagged AGO with endogenous AGO. Most studies also did not use semi-quantitative or quantitative techniques, further complicating the identification of candidates over background identifications that are not physiologically relevant. Finally, none of these studies has characterized the similarities and differences of cytoplasmic versus nuclear AGO2 complexes.

To identify nuclear interactions of AGO2, we performed a semi-quantitative proteomic study. All studies were performed in duplicate or triplicate. To further reduce detection of artifactual proteins and efficiently prioritize candidates for experimental validation, we compared results from samples obtained after purification of Flag-tagged AGO2 and endogenous AGO2. Studies were performed in the presence and absence of RNase. We observed conservation between key nuclear and cytoplasmic interactions, including interactions with AGO3 and all three TNRC6 paralogs. Interactions with TRBP were found in the cytoplasm, but not in the nucleus, consistent with strand loading being a cytoplasmic event.

## RESULTS

### Prioritizing candidate interacting partners for AGO2

**FIGURE 1.**
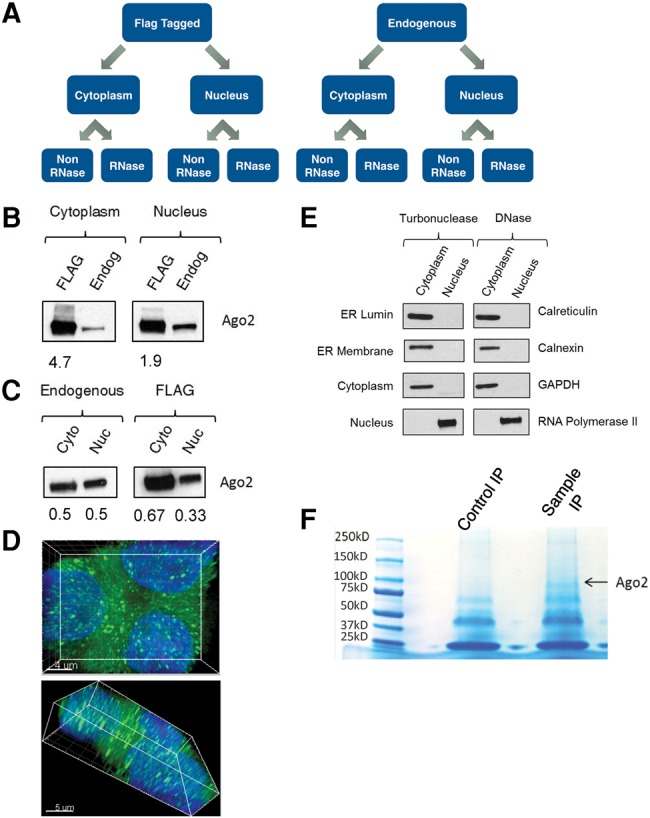
Characterization of mass spectrometry samples. (*A*) Schematic of experimental comparisons. (*B*) Western blot comparing levels of Flag-AGO2 with endogenous AGO2 in the cytoplasm and with expression in the nucleus. Cytoplasmic and nuclear extracts were separated by SDS-PAGE and blotted with anti-AGO2 antibody. Flag = AGO2 from Flag-tagged AGO2 stable line. Values are fold overexpression as compared to endogenous. (Endog) Endogenous AGO2 from T47D cells. (*C*) Western blot comparing expression of Flag-tagged AGO2 and endogenous AGO2 in the cytoplasm (*left*) and in the nucleus (*right*). Values are distribution levels between the cytoplasm and nucleus. (Cyto) Cytoplasm; (Nuc) nucleus. (*D*) Fluorescence images of Flag-AGO2. Green is Flag-AGO2, blue is the nucleus (stained with DAPI, 4′,6-diamidino-2-phenylindole). (*E*) Western blot analysis of cytoplasmic and nuclear fractions from RNase-treated and non-RNase-treated extracts of markers for nuclear, cytoplasmic, and ER proteins. Coomassie staining of Flag immunoprecipitations from cytoplasmic lysate prior to mass spectrometry. The control is using T47D cytoplasmic lysate, the sample is using Flag-AGO2 stable line cytoplasmic lysate. The band at ∼100 kDa is identified as AGO2.

We used mass spectral analysis to investigate interactions of AGO2. Large-scale proteomic analysis of interaction networks often leads to long lists of candidate proteins and it is important to prioritize the candidates for experimental validation. To facilitate identification of high priority candidates, we varied key experimental parameters and compared candidate lists. Our comparisons evaluate samples on three levels (Fig. 1A): (i) Flag-tagged AGO2 purified using an anti-Flag antibody versus endogenous AGO2 immunoprecipitated with an anti-AGO2 antibody; (ii) isolation from purified nuclei or cytoplasm; and (iii) samples isolated either with or without treatment with RNase.

For semi-quantitative proteomic analysis, we used the normalized spectral index (SINQ) method (Griffin et al. 2010). SINQ is a label-free proteomic quantitation method. Normalized spectral counts for each protein are calculated using many factors, including the number of peptides identified for the protein, the weight given for the assignment of each peptide to the protein, the number of spectrum matches for each peptide found in the protein, and the summed fragment ion-intensity for the assignment of an MS/MS spectrum to a peptide*.* This value is then normalized by the sum of all these values across all proteins in the data set, correcting for differences in protein loading between data sets. This result is then divided by the protein length, correcting for the propensity of longer proteins to produce a greater number of peptides than shorter proteins.

Normalized spectral counts can then be used to provide ratios between sample and control for each protein (Griffin et al. 2010; Trudgian et al. 2011a). Protein candidates characterized by a minimum spectral count of five as well as a minimum enrichment ratio of 5:1 for sample versus control were identified as top candidates. Data are presented as normalized spectral counts for each significant protein. To increase statistical rigor when prioritizing protein rankings, we also applied “Statistical Analysis of the Interactome” (SAINT) analysis.

### Expression and purification of Flag-tagged and endogenous AGO2

In this study we used both Flag-tagged AGO2 and antibodies against endogenous AGO2 in parallel experiments. The advantage of using anti-AGO2 antibody is that it can detect endogenous AGO2 and its partners at normal levels of AGO2 expression. The disadvantage is that using anti-AGO2 antibody runs the risk of cross-reactivity with AGO1, AGO3, or AGO4 proteins. The use of Flag-tagged AGO2 is advantageous because the interaction between the Flag epitope and the antibody is selective and maximizes the potential to detect interacting factors. One disadvantage is that introducing a tag may alter the ability of AGO to interact with some proteins (Oeffinger 2012). A second potential disadvantage is that tagged AGO2 may not be expressed at the same level as endogenous AGO2. Identifying candidates that are pulled down after both strategies would streamline identification of candidate interaction partners for experimental validation.

We used T47D cells because we had previously observed the involvement of AGO2 when promoter-targeted duplex RNAs were used to modulate gene transcription of the progesterone receptor in the nucleus of those cells (Janowski et al. 2005, 2007). We established T47D cells that stably express Flag-tagged AGO2. Western analysis of Flag-AGO2 cells using anti-AGO2 antibody suggests that AGO2 levels are approximately 4.7-fold higher than in the cytoplasm and 1.9-fold higher in the nucleus relative to endogenous AGO2 in T47D cells (Fig. 1B). The difference in expression of Flag-tagged and endogenous AGO2 underscores the value of including proteomic analysis of the endogenous protein.

In T47D cells the levels of endogenous AGO2 were approximately equal in the nucleus and cytoplasm (Fig. 1C). In the Flag-AGO2 stable line, cells have a distribution of 67% cytoplasmic and 33% nuclear AGO2. Our laboratory (Gagnon et al. 2014a) and others (Schraivogel and Meister 2014) have previous visualized endogenous AGO2 in cell nuclei and we used microscopy to detect nuclear Flag-tagged AGO2 (Fig. 1D).

### Purification of nuclear or cytoplasmic AGO2

The previous studies identifying AGO2 interacting partners focused on preparations from whole cells, ribosomal gradient fractions, or chromatin (Meister et al. 2005; Höck et al. 2007; Robb and Rana 2007; Landthaler et al. 2008; Weinmann et al. 2009; Ameyar-Zazoua et al. 2012; Frohn et al. 2012; Carissimi et al. 2015). One of our goals was to analyze cytoplasmic and nuclear interactions using extracts isolated from each compartment. Analysis of nuclear AGO2 requires rigorous isolation of cell nuclei. AGO2 is found in the endoplasmic reticulum (ER), which is contiguous with the nuclear envelope (Stalder et al. 2013). We have previously developed protocols for separating nuclei from ER (Gagnon et al. 2014b). Western blot analysis demonstrated that nuclear preparations lacked ER protein or cytoplasmic contaminants (Fig. 1E). Coomassie staining was routinely used as a quality control check to visualize an AGO2 protein band after immunoprecipitation to confirm adequate coverage for mass spectrometry (Fig. 1F).

### Cytoplasmic interactions of AGO2

**FIGURE 2.**
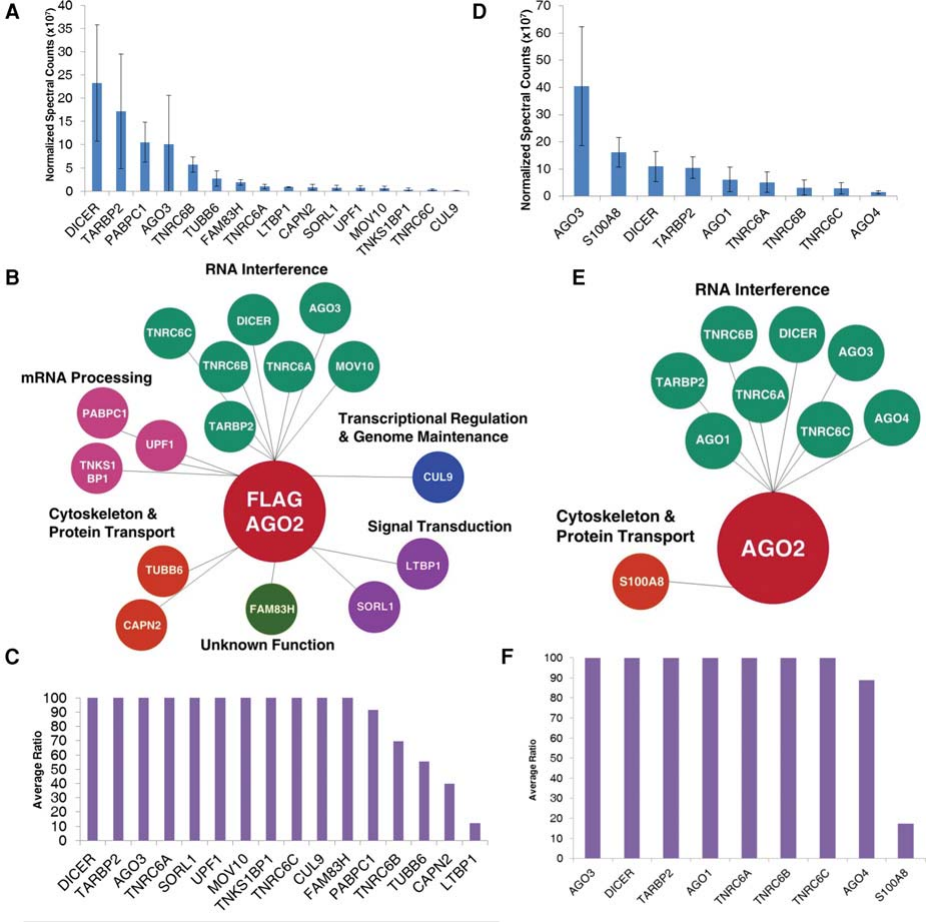
Identification of candidates from cytoplasmic immunoprecipitations (IPs) that had not been treated with RNase. (*A*) Normalized spectral counts of candidates identified from Flag-AGO2 IPs in the cytoplasm. *n* = 3. (*B*) Bubble plot of candidates identified from Flag-AGO2 IPs in the cytoplasm. Candidates are organized by function. (*C*) Average ratios of the normalized spectral counts between sample and control for candidates identified from Flag-AGO2 IPs in the cytoplasm. When no ratio was given (candidate was identified in sample only), a ratio of 100 was assigned. Ratios greater than 100 are represented at the cutoff. (*D*) Normalized spectral counts of candidates identified from endogenous AGO2 IPs in the cytoplasm. *n* = 3. (*E*) Bubble plot of candidates identified from endogenous AGO2 IPs in the cytoplasm. (*F*) Average ratios of the normalized spectral counts between sample and control for candidates identified from endogenous AGO2 IPs in the cytoplasm. When no ratio was given (candidate was identified in sample only), a ratio of 100 was assigned. Ratios greater than 100 are represented at the cutoff.

We began our analysis by examining the interactions of Flag-tagged AGO2 or endogenous AGO2 in isolated cytoplasmic extract from T47D cells (Fig. 2; Supplemental Tables 1, 2). For initial experiments, RNase was not added so that RNA-mediated interactions remained intact. Mass spectrometry revealed potential interactions between Flag-tagged AGO2 and proteins from several different protein families (Fig. 2A–C). In contrast to results obtained after use of anti-Flag purification, the only factors significantly detected after immunoprecipitation of endogenous AGO2 using anti-AGO2 antibody were proteins previously reported to be RNAi factors and the cytoskeletal protein S100A8 (Fig. 2D–F). S100A8 was much less selective for AGO2-immunoprecipitated sample relative to control IPs, with low enrichment ratios.

**FIGURE 3.**
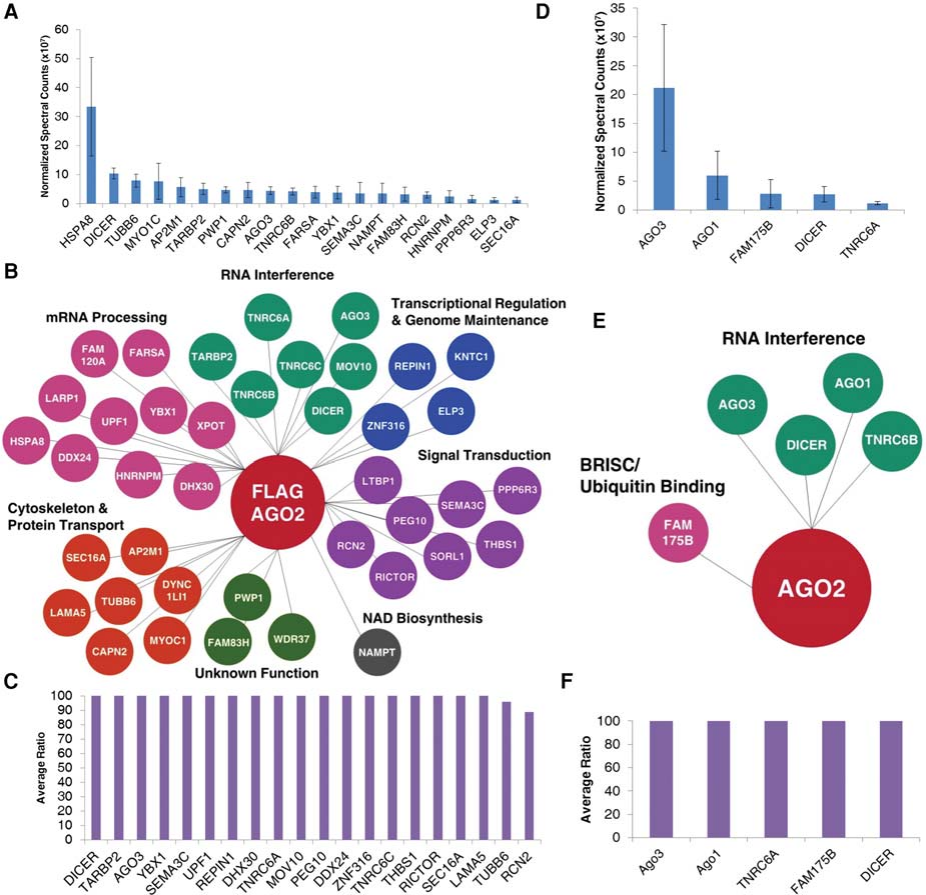
Identification of candidates from cytoplasmic immunoprecipitations (IPs) that had been treated with RNase. (*A*) Normalized spectral counts of top 20 candidates identified from Flag-AGO2 IPs in the cytoplasm. *n* = 3. (*B*) Bubble plot of candidates identified from Flag-AGO2 IPs in the cytoplasm. Candidates are organized by function. (*C*) Average ratios of the normalized spectral counts between sample and control for top 20 candidates (by ratio) identified from Flag-AGO2 IPs in the cytoplasm. When no ratio was given (candidate was identified in sample only), a ratio of 100 was assigned. A maximum cutoff of 100 was assigned to the graph. Ratios greater than 100 are represented at the cutoff. (*D*) Normalized spectral counts of top 20 candidates identified from endogenous AGO2 IPs in the cytoplasm. *n* = 3. (*E*) Bubble plot of candidates identified from endogenous AGO2 IPs in the cytoplasm. (*F*) Average ratios of the normalized spectral counts between sample and control for candidates identified from endogenous AGO2 IPs in the cytoplasm. When no ratio was given (candidate was identified in sample only), a ratio of 100 was assigned. Ratios greater than 100 are represented at the cutoff.

We subsequently extended our analysis to cytoplasmic samples that had been treated with RNase. After purification using Flag-AGO2, more than 40 proteins belonging to several functional groups were identified as candidate interacting partners (Fig. 3A–C; Supplemental Fig. S1; Supplemental Table 3). Many of these proteins possessed enrichment ratios relative to controls above 100:1. Several RNAi factors were identified, including Dicer, TNRC6A, TNRC6B, TRBP, and AGO3. In contrast to 30 candidates identified after immunoprecipitation of Flag-Ago2, only five candidates were identified after immunoprecipitation of endogenous AGO2 (Fig. 3D–F; Supplemental Table 4). Of these five candidates, four were RNAi factors, Dicer, TNRC6B, Ago1, and Ago3. All of these candidates had ratios above 100:1 or were not identified in the control sample. SAINT analysis revealed similar ranking of interacting partners from cell cytoplasm (Supplemental Fig. S2).

### Nuclear interactions of AGO2

**FIGURE 4.**
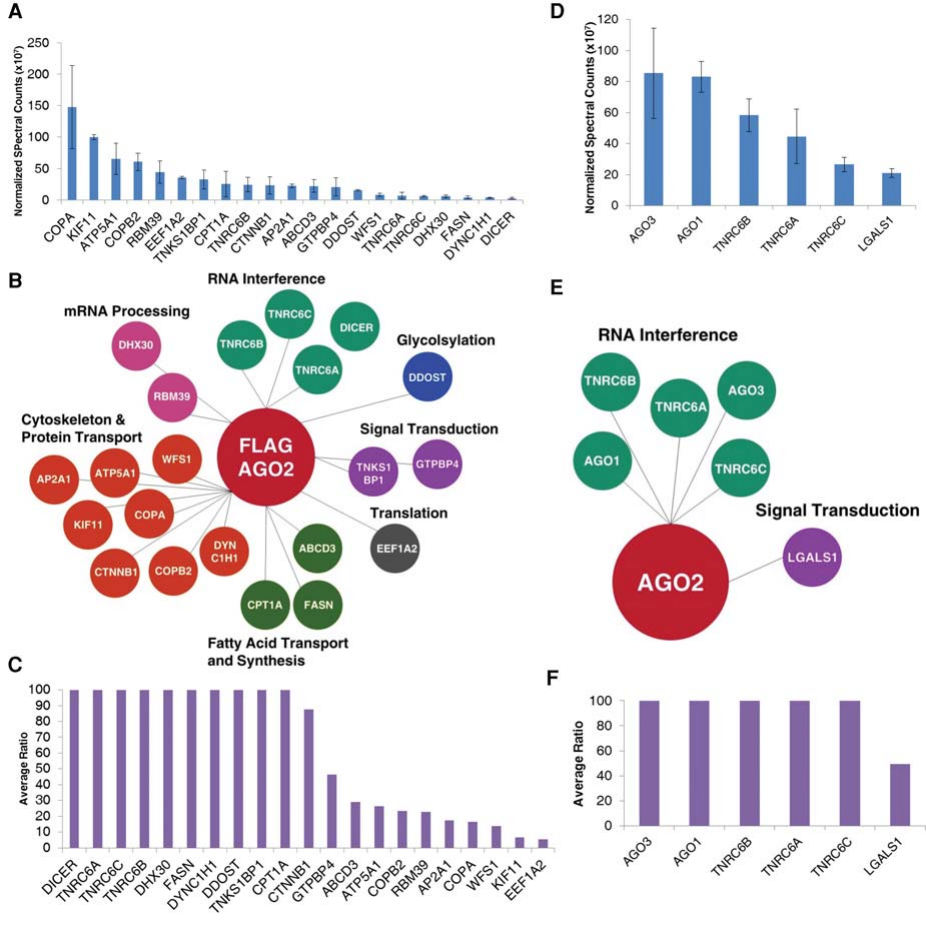
Identification of candidates from nuclear immunoprecipitations (IPs) that had not been treated with RNase. (*A*) Normalized spectral counts of candidates identified from Flag-AGO2 IPs in the nucleus. *n* = 2. (*B*) Bubble plot of candidates identified from Flag-AGO2 IPs in the nucleus. Candidates are organized by function. (*C*) Average ratios of the normalized spectral counts between sample and control for candidates identified from Flag-AGO2 IPs in the nucleus. When no ratio was given (candidate was identified in sample only), a ratio of 100 was assigned. A maximum cutoff of 100 was assigned to the graph. Ratios greater than 100 are represented at the cutoff. (*D*) Normalized spectral counts of candidates identified from endogenous AGO2 IPs in the nucleus. *n* = 3. (*E*) Bubble plot of candidates identified from endogenous AGO2 IPs in the nucleus. (*F*) Average ratios of the normalized spectral counts between sample and control for candidates identified from endogenous AGO2 IPs in the nucleus. When no ratio was given (candidate was identified in sample only), a ratio of 100 was assigned. Ratios greater than 100 are represented at the cutoff.

After completing analysis of cytoplasmic samples, we performed a parallel analysis to identify the interaction network of nuclear AGO2. After mass spectrometry of proteins associated with Flag-AGO2, we identified 21 proteins as candidate interacting partners (Fig. 4A–C). The top candidates possessed a wide array of functions, from apoptosis to RNA processing and included the RNAi factors Dicer, TNRC6A, TNRC6B, and TNRC6C. As previously noted for cytoplasmic samples, use of anti-AGO2 antibody for the immunoprecipitation led to detection of fewer candidate proteins. We identified interactions with the RNAi factors AGO3, AGO1, TNRC6A, TNRC6B, and TNRC6C as well as the signal transduction protein LGALS1 (Fig. 4D–F; Supplemental Table 7). Identification of LGALS1 was characterized by the fewest spectral counts and the lowest ratio of sample to background.

**FIGURE 5.**
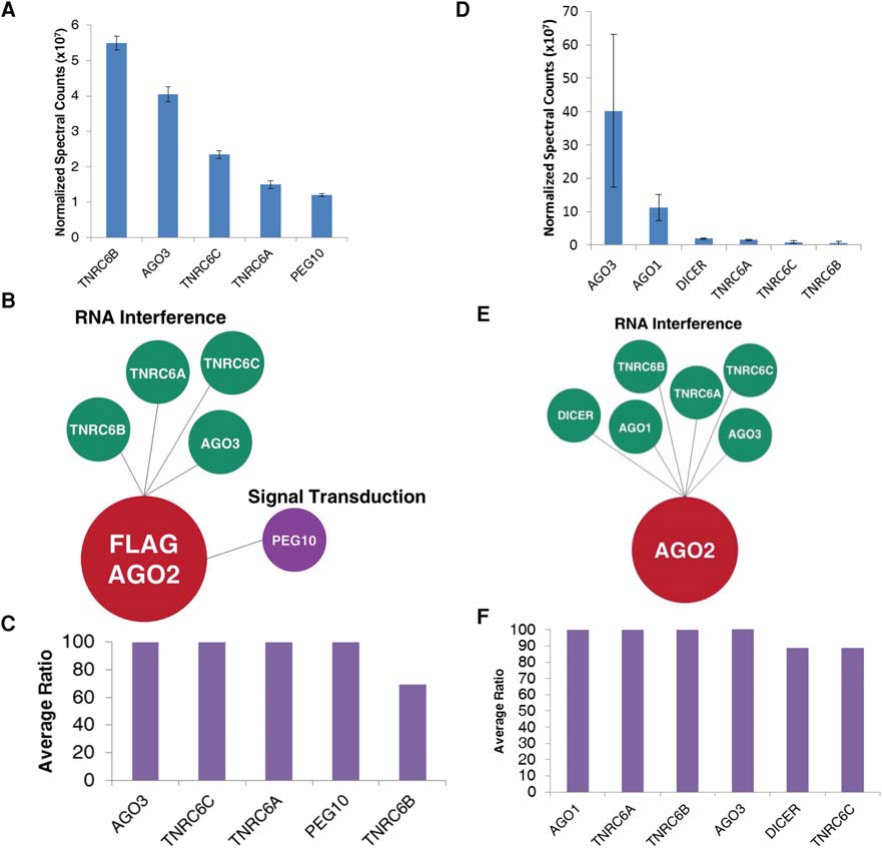
Identification of candidates from nuclear immunoprecipitations (IPs) that had been treated with RNase. (*A*) Normalized spectral counts of candidates identified from Flag-AGO2 IPs in the nucleus. *n* = 2. (*B*) Bubble plot of candidates identified from Flag-AGO2 IPs in the nucleus. Candidates are organized by function. (*C*) Average ratios of the normalized spectral counts between sample and control for candidates identified from Flag-AGO2 IPs in the nucleus. When no ratio was given (candidate was identified in sample only), a ratio of 100 was assigned. A maximum cutoff of 100 was assigned to the graph. Ratios greater than 100 are represented at the cutoff. (*D*) Normalized spectral counts of candidates identified from endogenous AGO2 IPs in the nucleus. *n* = 3. (*E*) Bubble plot of candidates identified from endogenous AGO2 IPs in the nucleus. Candidates are organized by function. (*F*) Average ratios of the normalized spectral counts between sample and control for candidates identified from endogenous AGO2 IPs in the nucleus. When no ratio was given (candidate was identified in sample only), a ratio of 100 was assigned. Ratios greater than 100 are represented at the cutoff.

We then examined the effect of treating nuclear samples with RNase. This treatment dramatically reduced the number of proteins recovered after Flag-tagged purification, from 22 to just five including RNAi factors and the signal transduction protein PEG10 (Fig. 5A–C; Supplemental Table 8). When we used an anti-AGO2 antibody, only RNAi factors were identified (Fig. 5D–F; Supplemental Table 9). All candidates isolated using either anti-Flag or anti-AGO2 purifications were characterized by high ratios of normalized spectral counts relative to control immunoprecipitations (Fig. 5C,F). SAINT analysis revealed similar candidate prioritization (Supplemental Fig. S3).

One concern with the identification of potential AGO2 interactions in nuclear samples is that they might be due to low-level contamination from the cytoplasm. Our Western analysis of nuclear and cytoplasmic fractions suggests that contamination is not extensive (Fig. 1E). Mass spectrometry, however, is sensitive and might detect contamination that escapes visualization on protein blots. To test this possibility, we compared the number of spectral counts detected for key RNAi factors in the extracts from nuclei and cytoplasm. Our analysis indicates that AGO3 TNRC6A, TNRC6B, and TNRC6C are detected with nuclear spectral counts similar to and in some cases greater than the spectral counts detected in the cytoplasm (Supplemental Figs. S2–S4). These data showing relatively high numbers of spectral counts in the nucleus relative to the cytoplasm suggest that the detected interactions are not due to trace contamination.

### Evaluating interactions with predicted protein partners

Our mass spectrometry experiments provided the data necessary to prioritize candidate interacting partners for further analysis. We compared lists of candidates generated after immunoprecipitation with anti-AGO2 antibody with lists generated after isolation using Flag-AGO2. Candidate proteins that were recovered by both purification schemes were validated by coimmunoprecipitation (co-IP). We also examined the potential for AGO2-protein interactions that may have had less strong support from our mass spectral data but that had intriguing functions or had been implicated in nuclear RNAi by others.

**FIGURE 6.**
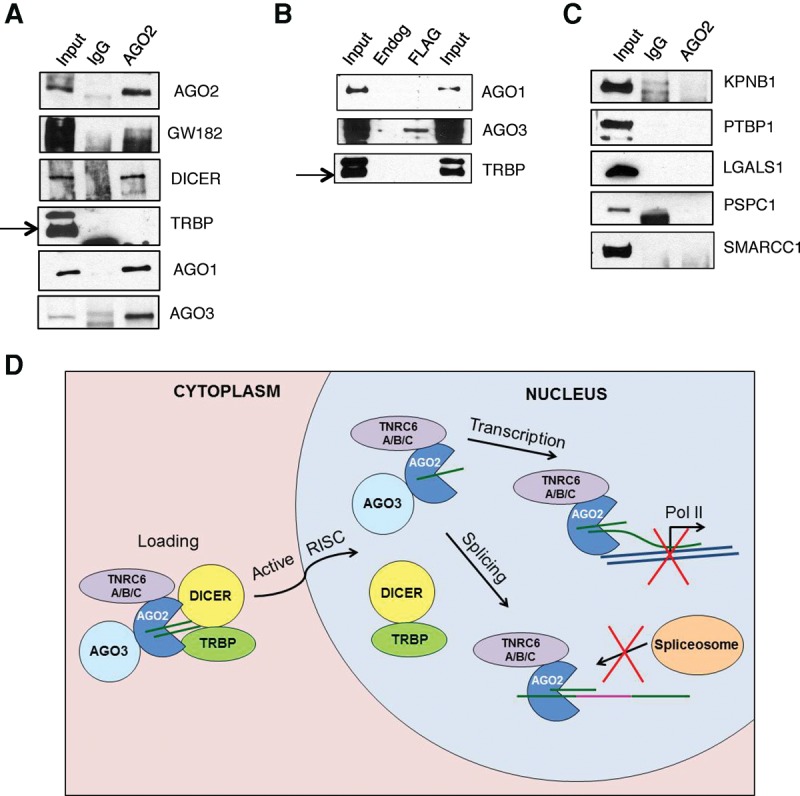
Coimmunoprecipitations of candidate interacting factors. (*A*) Co-IP of AGO2 with RNAi factors from the nucleus. AGO2 was immunoprecipitated versus an IgG control and interacting factors were blotted for with antibodies by Western blot. (*B*) Co-IP of Flag-AGO2 with interacting factors from the nucleus identified by mass spectrometry. Flag-AGO2 was immunoprecipitated from Flag-AGO2 stable line cells versus a Flag IP from endogenous T47D cells. Interacting factors were blotted for with antibodies by Western blot. (*C*) Co-IP of AGO2 with potential interacting factors from the nucleus. AGO2 was immunoprecipitated versus an IgG control and interacting factors were blotted for with antibodies by Western blot. (*D*) Model of nuclear AGO2 activity. AGO2 is bound by TRNC6 proteins and AGO3 in the cytoplasm. AGO2 is loaded by DICER, TRBP, and other loading factors. Active RISC is then shuttled into the nucleus. Interactions with DICER and TRBP are more transient in the nucleus. Loaded AGO2 and TNRC6 proteins can then alter transcription or splicing within the nucleus, depending on the small RNA bound by AGO2.

Interactions with TNRC6 and Dicer were confirmed by immunoprecipitation with anti-AGO2 antibody followed by Western analysis (Fig. 6A). Western analysis also supported an association between AGO2 and AGO3 (Fig. 6A,B). Consistent with our mass spectral data, no interaction was observed with TRBP after immunoprecipitation of nuclear extract (Fig. 6A). As a control, we showed that the anti-TRBP antibody could detect TRBP after immunoprecipitation of a cytoplasmic sample with anti-AGO2 antibody (Supplemental Fig. S5).

The potential for interaction between AGO1 and AGO2 was less clear. Our mass spectrometry indicated a potential interaction with AGO1 after immunoprecipitation with an anti-AGO2 antibody (Figs. 2D–F, 3D–F, 4D–F, 5D–F) but not when Flag-AGO2 was recovered using an anti-Flag antibody (Figs. 2A–C, 3A–C, 4A–C, 5A–C). Western analysis using anti-AGO1 antibody after immunoprecipitation using anti-AGO2 antibody appeared to detect AGO1 (Fig. 6A). A similar analysis of proteins recovered after isolation of Flag-AGO2 using an anti-Flag antibody did not reveal association between AGO2 and AGO1 (Fig. 6B). Taken together, the mass spectral data and Western analysis are most consistent with the conclusions that detection of an AGO1:AGO2 interaction is due to cross-reactivity between AGO1 and the anti-AGO2 antibody.

We also examined several potentially novel factors (Fig. 6C). LGALS1 is a protein that has known extracellular functions (Astorgues-Xerri et al. 2014) but was identified in the nuclear MS data as the only candidate that was not a known RNAi factor. It was not identified in the Flag-AGO2 MS, and it had the lowest ratio and spectral counts of the endogenous MS data set (Fig. 4D,F). LGALS1 was not detected with AGO2 after co-IP and Western analysis.

Several factors did not meet the cutoff standards used to identify high priority candidates but were examined because their biological functions are consistent with nuclear RNAi function or the transport of RNAi factors. PTBP1 is a protein involved in splicing (Ghetti et al. 1992) and was considered because it was close to the cutoff for candidate selection. KPNB1 is a karyopherin (Chi et al. 1995), a class of nuclear import proteins that might be involved in the transport of AGO2. We also considered the possibility that AGO2 may be interacting with nuclear bodies involved in RNA processing as it does in the cytoplasm. PSPC1, a paraspeckle protein (Fox et al. 2002), was regularly identified but at nearly the same frequency as in control samples. In spite of the potential for intriguing biological connections, PTBP1, KPNB1, and PSPC1 did not coimmunoprecipitate with AGO2 and were not validated as interacting factors (Fig. 6C).

We also tested the potential for AGO2 interactions with the protein SMARCC1 that had been identified in a whole cell study (Carissimi et al. 2015). SMARCC1 is an intriguing candidate because the protein is primarily nuclear and has functions in the SWI/SNF complex. Our cytoplasmic, nuclear, and whole cell (Supplemental Fig. S6; Supplemental Tables 9, 10) mass spectral analysis however, had not detected SMARCC1 as a candidate interacting protein. Co-IP using anti-AGO2 antibody for the pull-down from nuclear extract did not identify an interaction between AGO2 and SMARCC1 (Fig. 6C). SMARCC1 also failed to pull down AGO2 in a reciprocal Co-IP (Supplemental Fig. S7).

## DISCUSSION

Understanding the potential for RNAi in cell nuclei requires a more detailed knowledge of protein–protein interactions made by nuclear RNAi factors. In this study we focused on AGO2 because it is (i) a central factor for RNAi activity (Hammond et al. 2001; Rand et al. 2004; Joshua-Tor and Hannon 2011); (ii) present in mammalian cell nuclei (Robb et al. 2005; Ohrt et al. 2012; Gagnon et al. 2014a); and (iii) can modulate RNA-mediated control of transcription (Janowski et al. 2005; Li et al. 2006; Chu et al. 2010; Huang et al. 2010; Matsui et al. 2013) and splicing (Liu et al. 2012, 2015). RNA sequencing (RNA-seq) of nuclear RNAs that interact with AGO2 has revealed read clusters near intron–exon junctions and at gene promoters (Chu et al. 2015).

### Focusing candidate identification using data from orthogonal analyses

A primary challenge confronting the use of mass spectrometry data is prioritizing the selection of candidates for further testing (Trapp et al. 2014). We adopted orthogonal approaches to crosscheck mass spectrometry data and restrict the number of candidate proteins for subsequent validation. One data set was obtained using Flag-based purification of Flag-AGO2. The other data set was based on samples analyzed after purification of endogenous AGO2 using anti-AGO2 antibody. Candidates identified in common by the two strategies became the best candidates for further validation.

Flag purifications typically yielded many more candidates than purifications using an anti-AGO2 antibody (Figs. 2–4). This outcome may be because the Flag-AGO2 protein was overexpressed relative to endogenous AGO2. Alternatively, the Flag tag may be forming independent interactions with cellular proteins or altering the interactions made by AGO2. We note that our orthogonal analysis, while generating a shorter list of highly ranked candidates, provides no direct evidence that proteins identified in Flag-AGO pull-downs but not endogenous-AGO pull-downs are less likely to be biologically relevant interactors. Indeed, some of the top candidates from both Flag and anti-AGO purifications were the same proteins. In our experience, orthogonal screening was useful because it increased confidence in candidate selection for experimental validation, allowing resources to be directed more efficiently.

The evaluation of AGO1 as a candidate is an example of the advantage of incorporating orthogonal crosschecking into mass spectrometry. When purifying endogenous AGO2, we consistently identified AGO1 as an interacting partner. In contrast, AGO1 was not identified as an interacting partner of Flag-tagged AGO2. AGO2 and AGO1 have similar amino acid sequences, with 78.5% sequence homology. It is possible that the anti-AGO2 antibody used in these studies can also recognize AGO1 and cause AGO1 to be detected as a candidate interaction.

### Stable interactions of RNAi factors are conserved between nucleus and cytoplasm

In the cytoplasm, when either endogenous AGO2 or Flag-tagged AGO2 extracts were not treated with RNase, we detected only six proteins above our cut-off levels, TNRC6A, TNRC6B, TNRC6C, Dicer, AGO3, and TRBP2 (Supplemental Fig. S8A). When AGO2 or Flag-tagged extracts were treated with RNase, three were identified in common, AGO3, Dicer, and TNRC6B (Supplemental Fig. S8B). These data from two different modes of purification and two different nuclease treatments suggest that the central cytoplasmic complex includes AGO2, Dicer, and TNRC6B. The other factors, TNRC6A, TNRC6C, AGO3, and TRBP2 may dissociate or bind less strongly upon treatment with RNase H.

When we compared nuclear extract data from the two isolation strategies, we identified three common interacting factors, TNRC6A, TNRC6B, and TNRC6C (Supplemental Fig. S8C). After RNase treatment of nuclear samples, we identified the same central RNAi factors and also AGO3 (Supplemental Fig. S8D). These data suggest that stable interactions of AGO2 within the nucleus are limited to a handful of proteins, and that interactions with TNRC6 family members are not affected by treatment with TurboNuclease.

Our comparison of results from nuclear and cytoplasmic samples reveals both similarities and differences. For every sample, regardless of cellular origin, we detected association with at least one TNRC6 family member. These data suggest that a stable RNAi complex of AGO2 and TNRC6 paralogs is conserved between the cytoplasm and the nucleus.

Association with Dicer and TRBP was detected only in the cytoplasm. The role of TRBP in RISC loading is to stabilize the Dicer/Ago interaction and aid in guide strand loading (Wang et al. 2009; Wilson et al. 2015). Dicer and TRBP are involved in strand loading and differences between their association with AGO2 in the cytoplasm and nucleus supports prior findings that AGO loading is a cytoplasmic process (Gagnon et al. 2014a).

We had previously shown that TRBP was present in mammalian cell nuclei and could be detected in complex with AGO2 (Gagnon et al. 2014a). The interaction between TRBP and Ago2 is believed to be more transient than the interaction between Ago2 and Dicer (Wang et al. 2009), and it is possible that it is too transient in the nucleus to be identified by our purification protocols used to prepare samples for mass spectrometry.

TNRC6A/GW182 is a well-known partner of AGO2. It is known to stabilize the AGO2–miRNA–mRNA interactions, as well as localizing AGO2 to P-bodies in the cytoplasm (Pfaff and Meister 2013; Pfaff et al. 2013). The three TNRC6 paralogs (A, B, and C) have some similarities—they each bind AGO2 and localize the complex to P-bodies (Lazzaretti et al. 2009). They all share the same domain organization and have two unstructured GW-repeat regions (Ding and Han 2007). We have previously observed that simultaneous knockdown of all three TNRC6 proteins blocked RNA-mediated activation of cyclooxygenase-2 expression while knocking down TNRC6A alone was insufficient (Matsui et al. 2013).

Ui-Tei and colleagues have suggested that TNRC6 proteins direct AGO2 shuttling between the nucleus and cytoplasm (Nishi et al. 2013). AGO2 does not possess an obvious nuclear import sequence, and the nuclear localization and export sequences within TNRC6 may be critical for guiding the complex (Nishi et al. 2013). Meister and colleagues, however, have recently suggested that nuclear transport of AGO2 and TNRC6 paralogs depend on different and possibly redundant transport pathways (Schraivogel and Meister 2014). They have gone on to show that TNRC6 proteins along with AGO2 use the importin pathway, and that nuclear localization of both can be codependent (Schraivogel et al. 2015).

Our isolations of AGO2 do not use crosslinking. The interactions that we detect must be stable enough to survive multiple washings and desalting using size exclusion chromatography. Further studies will be necessary to fully explore more transitory interactions or interactions that might be made indirectly through TRNC6 paralogs rather than direct association with AGO2.

### Protein interactions and the mechanism of nuclear RNAi

Our findings allow us to refine the model for the action of AGO2 in the cytoplasm and nucleus (Fig. 6D). AGO2 forms a stable complex with TNRC6 family members in both compartments. We also detect association with AGO3 in both compartments. AGO3 has been reported to stabilize the level of Alu RNAs (Hu et al. 2012) and the association that we detect may reflect a broader role for AGO3 in RNA-mediated control of RNA metabolism. AGO3 has also been reported to partially compensate for depletion of AGO2 (Ruda et al. 2014) and to affect maturation of let-7a miRNA (Winter and Diederichs 2013).

Previous studies have suggested that strand loading occurs in the cytoplasm (Ohrt et al. 2012; Gagnon et al. 2014a). Our data are consistent with that conclusion and suggest that the AGO2:TNRC6 complex interacts with Dicer and TRBP in the cytoplasm. Loaded complex must then be imported into cell nuclei, possibly through interactions with Importin 8 (Weinmann et al. 2009). The guide strand RNA:AGO2:TNRC6 complex might then hybridize to nascent transcripts to affect critical processes like transcription (Matsui et al. 2013) or pre-mRNA splicing (Liu et al. 2012).

### Conclusion

Mass spectrometry is a powerful tool for studying protein interactions but is challenged to efficiently discriminate between high priority candidate interactions and background. Orthogonal methods for sample isolation allowed us to crosscheck results and prioritize candidate interactions. A stable complex between AGO2 and TNRC6 family paralogs is conserved between the cytoplasm and the nucleus. Interactions with Dicer and TRBP are not consistently observed in nuclear samples, consistent with strand loading and processing occurring in the cytoplasm. The finding that several protein interacting partners of the RNAi complex are conserved in the nucleus supports the hypothesis that nuclear RNAi has the capacity to drive recognition of nuclear RNA sequences and affect nuclear RNA metabolism and expression.

We do not exclude the possibility that other proteins form significant interactions with AGO2 because interactions that are less stable but biologically significant may be undetected by mass spectrometry. Important interactions with nuclear proteins that are mediated through TNRC6 family proteins or Dicer may also contribute to function but might not be apparent when AGO2 is used as the bait protein. Now that AGO2 has been shown to form a limited number of stable interactions that can be detected by mass spectrometry, it may be useful to shift attention to the next shell of interacting partners to gain further insight into how RNAi factors function in splicing and transcriptional regulation.

## MATERIALS AND METHODS

### Cell culture

T47D cells (ATCC) were cultured in RPMI medium supplemented with 10% FBS, 0.5% NEAA, 20 µg/mL insulin, 10 mM pH 7.0–7.6 HEPES, and 1 mM sodium pyruvate. T47D cells stably expressing Flag-HA-tagged AGO2 were cultured identically to T47D cells but media supplemented with 0.2 mg/mL G418. All cells were grown at 37°C in 5% CO_2_.

The Flag-HA-tagged AGO2 stable cell line was developed by transfecting T47D cells with the (corrected) pIRESnew-Flag/HA AGO2 plasmid (Addgene, 10822) and Lipofectamine 2000 (Life Technologies, 11668019). Cells were treated with 500 µg/mL G418 for 2 wk. Colonies were picked with 5% trypsin and transferred to 24-well dishes. Colonies were tested for expression of Flag-HA AGO2 by Western blot with anti-HA and anti-AGO2 antibodies. Colony labeled “Colony 1” was used for this study.

### Extract preparation

Purification of cytoplasmic and nuclear extract from T47D or Flag-AGO2 cells was performed as described previously (Gagnon et al. 2014b) with a few modifications. Rather than using sonication to lyse nuclei, a 27-gauge needle was used. For RNase extracts, TurboNuclease was added at a 1:10,000 dilution (Accelagen, N0103M) to the extract and incubated for 30 min at room temperature. For non-RNase-treated nuclear extracts, DNase was added at a 1:40 dilution (Worthington, LS006353) and incubated at 37°C for 30 min.

### Extract analysis

Western blots to determine the purity of nuclear and cytoplasmic fractions from TurboNuclease or DNase-treated T47D or Flag-AGO2 cells were performed as before (Gagnon et al. 2014b). For comparing nuclear and cytoplasmic fractions by Western blot, the same cell equivalents of extract were separated by electrophoresis (one-half the volume of nuclear extract per one volume of cytoplasmic). Blocked Western blot membranes (Hybond-C Extra, GE Healthcare Life Sciences) were incubated with the following primary antibodies for 16 h at 4°C in PBST (PBS + 0.05% TWEEN-20) + 5% milk with rocking: anti-AGO2 at 1:1000 (Abcam, ab57113), anti-Calreticulin at 1:1000 (Cell Signalling, 2891S), anti-Histone H3 at 1:10,000 (Abcam, ab1791), anti-Lamin A/C at 1:1500 (Abcam, ab8984), anti-GAPDH at 1:600 (Abcam, ab9484), anti-RNA polymerase II at 1:4000 (Millipore, 05-623).

### Immunoprecipitation

For immunoprecipitations of endogenous AGO2 in T47D extracts, either anti-AGO2 (Abcam, ab57113) or mouse IgG antibody (Millipore, 12-371) was cross-linked to Protein A Plus/Protein G resin. Antibody was incubated with resin (65 µg/mL resin) in coupling buffer (10 mM sodium phosphate, pH 7, 75 mM NaCl) with rocking at room temperature for 2 h. Resin was washed 3× with coupling buffer. Of note, 0.4 mM DSS was added to resin and incubated with rocking for 30 min at room temperature. The reaction was quenched with Tris–HCl, pH 8.0 and resin was resuspended in IP-EQ buffer (50 mM Tris–HCl, pH 7.4, 2 mM MgCl_2_, 150 mM KCl, 0.05% NP-40) for use in experiments.

Immunoprecipitations for mass spectrometry analysis were performed in either T47D or Flag-AGO2 cytoplasmic or nuclear extracts. Mouse IgG conjugated resin was the control for T47D extracts. Flag conjugated resin (Sigma, A2220) was incubated with extract from Flag-AGO2 stable line cells or incubated with extract from T47D cells lacking Flag-AGO2 as the control. For each extract, a starting protein concentration of 15 mg was pre-cleared using mouse IgG conjugated Agarose (Sigma, A0919), rocking at room temperature for 30 min. Extracts were transferred to 250 µL of specific-antibody bound resin and incubated overnight at 4°C with rocking. Resin was washed 6× with IP_300_ Buffer (20 mM Tris–HCl, pH 7.4, 2 mM MgCl_2_, 300 mM NaCl, 0.05% NP-40), and proteins were eluted with 250 µL of 1× SDS loading buffer. To eliminate excess salt, elutions were run through Illustra Nap-5 desalting columns (GE Healthcare, 17-0853-02). To concentrate samples, elutions were run through Amicon Ultra-0.5 mL centrifugal protein concentrators (Millipore, UFC901024). Elutions were run 15 mm into SDS-PAGE gels, Coomassie stained with GelCode Blue (Thermo, 24592), and each lane was cut into three 5-mm slices. The gel slices were submitted to the UT Southwestern Proteomics Core for further analysis.

### Mass spectrometry

Gel slices were digested overnight with trypsin (Promega) following reduction and alkylation with DTT and iodoacetamide (Sigma-Aldrich). Following solid-phase extraction cleanup with Oasis HLB plates (Waters), the resulting samples were analyzed by LC/MS/MS using an Orbitrap Elite, Q Exactive, or Q Exactive Plus mass spectrometer (Thermo Electron) coupled to an Ultimate 3000 RSLC-Nano liquid chromatography system (Dionex). Samples were injected onto either a 180-µm i.d., 15-cm-long column packed in-house with a reverse-phase material ReproSil-Pur C18-AQ, 1.9-µm resin (Dr. Maisch, GmbH), or a 75-µm i.d., 50-cm-long Easy Spray column (Thermo). Peptides were eluted with a gradient from 1% to 28% buffer B over 40 min (180 µm column) or 60 min (75 µm column). Buffer A contained 2% (v/v) acetonitrile (ACN) and 0.1% formic acid in water, and buffer B contained 80% (v/v) ACN, 10% (v/v) trifluoroethanol, and 0.08% formic acid in water. The mass spectrometer acquired up to 20 MS/MS spectra for each full spectrum acquired.

Cytoplasmic samples as well as two replicates of endogenous Nuclear RNase treatment samples were run on the Q Exactive (QE), one replicate of endogenous Nuclear RNase treatment samples were run on the Q Exactive Plus (QE+), and Flag Nuclear non-RNase and endogenous nuclear non-RNase treatment samples were run on the Orbitrap Elite (OTE). Instrument choice was based on mass spectrometer availability at the time of analysis.

Raw MS data files were converted to a peak list format and analyzed using the central proteomics facilities pipeline (CPFP), version 2.0.3 (Trudgian et al. 2010; Trudgian and Mirzaei 2012). Peptide identification was performed using the X!Tandem (Craig and Beavis 2004) and open MS search algorithm (OMSSA) (Geer et al. 2004) search engines against the human protein database from Uniprot, with stable contaminants and reversed decoy sequences appended (Elias and Gygi 2007). Fragment and precursor tolerances of 20 ppm and 0.5 Da were specified, and three missed cleavages were allowed. Carbamidomethylation of Cys was set as a fixed modification and oxidation of Met was set as a variable modification. Label-free quantitation of proteins across samples was performed using SINQ normalized spectral index software (Trudgian et al. 2011b).

### Data analysis

The standard spectral count cutoff for SINQ analysis is a spectral count of three or higher. To increase the stringency of the analysis, a cutoff of five spectral counts was used for all data sets. The second layer of analysis is through the ratios of sample to control. For this, a ratio cutoff of 5:1, respectively, was set. All samples were compared against all controls. The endogenous samples were run in triplicate and needed to meet the cutoff criteria in at least two of three experiments to be considered. Flag-AGO2 samples were run in duplicate and therefore had to meet the criteria in both data sets to be considered. Replicate data were obtained from independently grown cell cultures and independent purifications of AGO2 bound proteins.

In addition to the cutoff of at least five spectral counts to consider a prey protein as a true interactor with AGO2 bait, we also used the Significance Analysis of INTeractome (SAINT) program (Choi et al. 2011, 2012a, b), which assigns confidence scores to prey-bait interactions from the affinity purification–mass spectrometry data. The protein lists generated from the CPFP analysis (as explained above) were converted to SAINT-compatible tables for analysis. SAINT uses the spectral count data (Choi et al. 2011) to score these protein–protein interactions, and uses the negative control samples to model the spectral count distribution for false interactions, thus making the method a semi-supervised approach. A list of bait–prey protein interactions at a 1% false discovery rate (FDR) was generated to identify high-confidence protein interactors with AGO2 in each of the data sets analyzed (Supplemental Figs. S2, S3). Because three different instruments (QE, QE+, and OTE) were used for mass spectrometry, we performed principle component analysis to ensure that identification of candidate proteins was not instrument-dependent. We did not observe data clusters that are instrument-dependent. Conversely, we did observe that replicates arising from the same condition do cluster together even if the data were obtained from different instruments.

### Immunofluorescence

T47D cells (ATCC) and T47D cells stably expressing Flag-HA-tagged AGO2 were seeded on 35-mm dishes with a 14-mm glass bottom overnight. Cells were then fixed with 4% paraformaldehyde for 15 min, washed with PBS three times, and then permeabilized with 70% ethanol and 0.5% Triton X-100. Cells on the glass bottom were incubated with anti-AGO2 antibody (Wako; 1:50) in PBS/1% normal goat serum (NGS). Cells were washed with PBS three times and then incubated with Alexa Fluor 488 goat anti-mouse IgG antibody in PBS/1% NGS (for AGO2) for 1 h. Cells were washed with PBS three times and then covered with mounting medium with 4′,6-diamidino-2-phenylindole (DAPI). Cells were imaged by wide-field epifluorescence microscopy and images were processed by blind deconvolution with AutoQuant X3 (Media Cybernetics). Using Imaris (Bitplane), Z-sections from the middle of the cells (10 image slices; interval: 0.2 µm) were exported, as well as stacked and projected in three dimensions.

### Coimmunoprecipitations

For coimmunoprecipitations, 300 µg of starting protein was used per sample. Extract was precleared with mouse IgG conjugated resin (30 µL of 50% slurry) for 30 min at 4°C. Samples were then incubated with 3 µg of antibody for 1.5–2 h at 4°C. Forty-five micrograms of Protein G plus Protein A resin was added and incubated for another hour. For Flag IPs, 30 µL of a 50% slurry of Flag conjugated resin was added to the extract after preclear and incubated for 2.5–3 h. Samples were washed 6× with IP_300_ Buffer (20 mM Tris–HCl, pH 7.4, 2 mM MgCl_2_, 300 mM NaCl, 0.05% NP-40) with 5 min incubations and eluted with 95°C 1×SDS for 5 min with shaking. Samples were loaded onto 4%–20% SDS-PAGE gels and Western blots were performed. Antibodies used: GW182 (Bethyl, A302-329A); DICER (Abcam, ab14601); TRBP (Abcam, ab72110); AGO1 (Cell Signaling, D84G10); AGO3 (Sigma, SAB4200112); KPNB1 (Bethyl, A300-482A); PTBP1 (Abcam, ab5642); LGALS1 (Cell Signaling, 5418S); PSPC1 (Santa Cruz, SC84576); SMARCC1 (Abcam, ab72502).

## SUPPLEMENTAL MATERIAL

Supplemental material is available for this article.

## Supplementary Material

Supplemental Material
