# Supplementary material for: Stable association of RNAi machinery is conserved between the cytoplasm and nucleus of human cells
Source: RNA. 2016 Jul;22(7):1085–98. doi: 10.1261/rna.056499.116 (PMC4911916; doi:10.1261/rna.056499.116)
Supplement: Supplemental Material [file supp_22_7_1085__index.html]

Stable association of RNAi machinery is conserved between the cytoplasm and nucleus of human cells — Stable association of RNAi machinery is conserved between the cytoplasm and nucleus of human cells — Supplemental Material 

# Stable association of RNAi machinery is conserved between the cytoplasm and nucleus of human cells

## Supplemental Material

- Supplementary\_Tables.xlsx
